# Recombinant ferritins for multimodal nanomedicine

**DOI:** 10.1080/14756366.2023.2219868

**Published:** 2023-06-01

**Authors:** Yihao Li, Haoyu Gao, Eugenie Nepovimova, Qinghua Wu, Vojtech Adam, Kamil Kuca

**Affiliations:** a College of Life Science, Yangtze University, Jingzhou, China; b Department of Chemistry, Faculty of Science, University of Hradec Kralove, Hradec Králové, Czech Republic; c Department of Chemistry and Biochemistry, Mendel University in Brno, Brno, Czech Republic; d Biomedical Research Center, University Hospital Hradec Kralove, Hradec Kralove, Czech Republic

**Keywords:** Ferritin, recombinant protein, nanovaccine, nanozymes, nanocarrier

## Abstract

In all living organisms, ferritins are a group of proteins important for maintaining iron homeostasis. Increasing amount of studies has shown that recombinant ferritins can be widely used in multimodal nanomedicine, especially for anticancer treatment and vaccination. Recombinant particles prepared by fusing viral proteins and ferritin subunits produce a better immune response and higher antibody titres. Moreover, actively-targeted ferritin nanoparticles can recognise receptors and deliver natural or chemical drugs specifically to the tumour tissue. In addition, ferritin-linked or loaded with contrast agents or fluorescent dyes can be used as multimodal particles useful cancer theranostics. In this review, we fully summarised the unitisation of recombinant ferritins in multimodal nanomedicine. The research progress of using recombinant ferritins as nanovaccines, nanozymes, and bioengineered nanocarriers for targeted therapy and bioimaging is emphasised.

## Introduction

Ferritins (Ftn) are a class of important iron storage proteins widely present in all organisms that use Ftn for maintaining iron homeostasis^1^. Ftns usually consist of a spherical cage of 24 identical or heterologous H-and L-subunits^2^. Of these, the H-subunit provides rapid detoxification of iron through its inherent ferroxidase activity and the L-subunit promotes iron nucleation, mineralisation, and long-term storage^3^. As a natural polymeric biomaterial, Ftn has the advantage of good biocompatibility, biodegradability, and a long plasma half-life^4^. Noteworthy, Ftn is an important disease biomarker. Serum Ftn is routinely used in clinical medicine as a surrogate indicator of iron storage in the body, where low levels indicate iron deficiency^5^. Also, an inflammation or infection can dramatically alter serum Ftn levels^6^. In the pancreas, Ftn deposits can impair cells function and induce diabetes. Moreover, it can also lead to hypogonadism and hyperpigmentation in the pituitary gland and skin, respectively^7^. There are some hereditary ferritinopathy and neuroferritinopathy associated with iron accumulation, such as amyotrophic lateral sclerosis^8^, restless legs syndrome^9^, and Alzheimer’s disease^10^. High levels of serum Ftn are a marker of poor prognosis in restless legs syndrome.

With the development of recombinant DNA and protein engineering technologies, recombinant proteins have also shown unique potential for use in the biomedical research^11^. In recent years, there is a growing interest in Ftn-based vaccines^12^. The reversible assembly of subunits of Ftn using gene fusion SARS-CoV-2 vaccine^13^, which is in phase I clinical trials^14^. In addition, HIV and influenza vaccines have also been successfully designed^15–16^.

Besides use of Ftn in vaccination research, removal of the iron from Ftn internal cavity results in a hollow Ftn cage structure that can be loaded with variety of bioactive compounds and possesses many advantages as a carrier for cancer-targeted drugs^17^. For example, Ftn loaded with doxorubicin (Dox) finctionalized with polyethylene glycol (PEG) preferentially crossed the airway mucus barrier and released Dox uniformly into the orthotopic lung tumour tissue, increasing the survival rate of mice by up to 50%^18^. Additionally, loading of siRNA effectors into Ftn effectively prevents their degradation and avoids multiple drug administration^19^. Hepatocellular carcinoma-targeted Ftn (HccFtn) can effectively load six times the dose of free Dox used Carcinoma-targeted Ftn can slowly released and showed harmless to the healthy hepatocytes^20^. Moreover, Ftns can be employed as promising modalities in the rapidly developing filed of nanozymes. Engineered recombinant Ftns are an ideal tools to protect artificial metalloenzymes from harsh and complex *in vivo* environments, as has been shown in case of metallozymes loaded into Ftn. The loading did not alter peroxidase activity of metallozyme and improved generation of intracellular oxygen, thus proved useful for nanozyme-based anticancer therapy^21^. Although intratumoral administration of Ftn exhibits some advantages, uncontrollable proper assembly between recombinant protein subunits and heterogeneity of particles lead to limited clinical application of recombinant Ftn.

In this manuscript, we systematically reviewed the functional structure of Ftn, the research progress and future prospects of recombinant Ftn used in multimodal nanomedicine. We particularly focus on the research progress in the field of nanovaccines, nanozymes, bioengineered nanocarriers for targeted drug delivery and diagnostic imaging. We provide examples of selected applications of Ftn and summarise the advantages of Ftn in the field of nanomedical research, with the aim of providing a reference for the current and future medical research on recombinant Ftn.

## Ferritin utilisation in nanovaccine

The modification of the different subunits of Ftn nanoparticles by genetic engineering not only allows the introduction of antigens onto the surface of Ftn, but also ensures a stable and autonomous loading of various types of cargoes into the cavity of Ftn^22^. Using Ftn as vehicle, various vaccine candidates against different types of pathogens are currently in clinical trials H2 influenza^23^, or SARS-CoV-2 vaccines^24^.

### Ferritin induces high concentrations of neutralising antibodies

**Figure 1.**
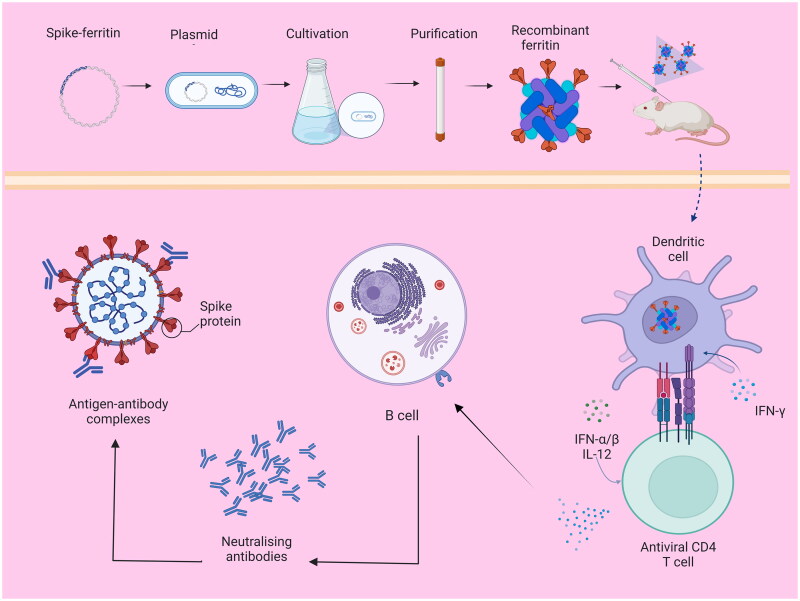
Recombinant ferritin induces high levels of neutralising antibodies.

Ftn-based nanoparticles designed for vaccination against SARS-COV-2/S^25^, influenzas and respiratory syncytial virus (RSV) induced high levels of specific antibodies in animals^26^ (Figure 1). A/Singapore/1/1957 haemagglutinin (HA) genetically fused to the *Helicobacter pylori*-derived Ftn (HP-Ftn) was shown to induce a broadly neutralising antibody response against influenza type 1 viruses (including seasonal H1 and avian H5 subtypes) ^23^. Furthermore, novel Coronavirus fusion Ftn was used to induce the production of neutralising antibodies in mice, and the induced antibody levels were twice higher than those of plasma donors in the recovery phase of SARS-COV-2^24^. Furthermore, the fusion of HP-Ftn with Epstein-Bar virus (EBV) surface glycoprotein can stimulate mice and primates to produce specific neutralising antibodies higher than those induced by an EBV glycoprotein only^27^. The fusion Ftn nanoparticles produced by fusing the HP-Ftn with the H1N1 influenza virus HA induced a rapid production of neutralising antibodies in mice that were more than 10-fold more potent than those produced by the normal vaccine and even were able to neutralise different subtypes of the influenza virus^28^. As the H- and L- chains of Ftn can be combined with different antigens to prepare recombinant vaccination particles containing two or more antigens, Ftn also enables facile development of multivalent nanovaccines^29^. *Staphylococcus aureu*s (SA) alpha-hemolysin is a frequent cause of deleterious cytotoxic effects of SA infections^30^. Therefore, a synthetic DNA encoding Hla121-138 was fused to the CDS of Ftn at the N-terminus, cloned into the pET-28A plasmid and introduced into BL21 (DE3) cells to express an epitope-based nanoparticle (EpNP) that immunologically induced potent haemolytic neutralising antibodies and conferred significant protection in a mouse model of SA skin infection^31^.

**Figure 2.**
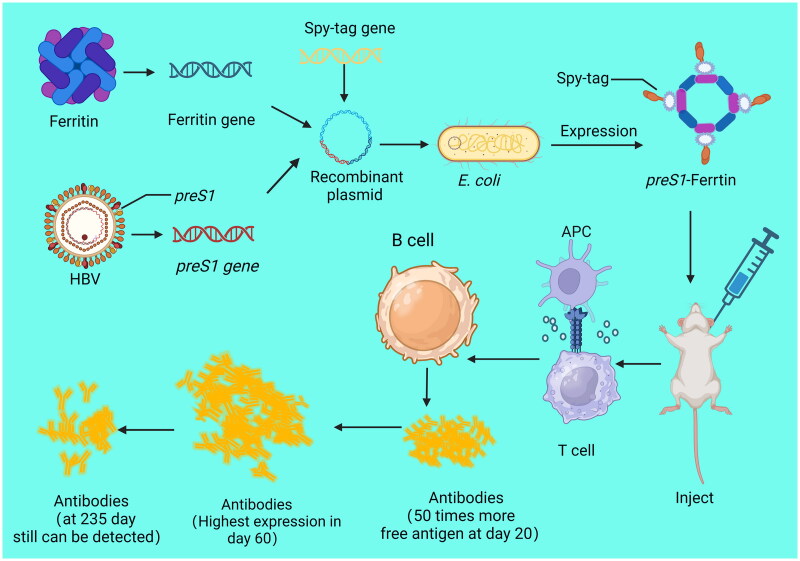
Long-term efficacy of ferritin nanovaccine.

Additionally, *preS1* is an antigenic protein on the surface of HBV. preS1 on the surface of recombinant Ftn actively targets SIGNR1+ macrophages and SIGNR1+ dendritic cells in lymph nodes and promotes the activation of B cells and T cells^32^ (Figure 2). Importantly, it is worth to note that Ftn can stimulate bone marrow dendritic cell maturation via the activated TLR4/NF-κB pathway, which in turn promotes T cell proliferation and differentiation. ct of Ftn nanovaccines may also be related to repeatedly display the target antigen^32^^,^^33^.

### Molecular size and shape affect the efficacy of recombinant protein vaccine

The immunological effect of antigenic peptides is related to their molecular size and shape. For example, when the RSV protein of enterovirus was presented the surface of Ftn, the antibody titres induced in mice were higher. Within a certain range, the immune effect increases with the antigenic peptide molecule^34^. *H. pylori*-bullfrog hybrids carrying residues 2–9 at the N-terminal end of bullfrog L-chain Ftn allow the formation of radially prominent tails that improve antigen presentation in many Ftn-based vaccine candidates and further enhance the effectiveness of immunisation^13^.

Viral stinger protein particles are recognised by immune cells to induce specific antibody production. By gene fusion techniques that allow the HA gene to be located just at the N-terminus of Ftn around the triple fold axis, HA monomers interact and are stable and allow conformation-dependent oligomerization of the trimeric antigen^35^. In particular, the Receptor binding domain (RBD) of the S-protein structural domain is directly involved in host receptor recognition, and amino acid variants in this region lead to changes in the genophilic and infectious properties of the coronaviruses, like SARS virus, enters cells by recognising the human angiotensin 2 (ACE2) protein of the human host^36^. RBD-based subunit vaccines were designed to provide the basis for new vaccine therapies against the new coronaviruses (nCoV) ^12^^,^^37^, entry is achieved by binding of coronavirus spike proteins to human ACE2 receptors. Spike-ACE2 interactions are mediated through the receptor RBD of spike proteins^38^. A fusion protein of H1 HA stabilized-stem on Ftn nanoparticle (H1-SS-NP) can induced passive transfer of immunoglobulins protected mice from lethal doses of *H5N1* virus killing^13^. The Ftn vaccine induces long-term antibodies with good results. three potentially linear sequences in the Yanaka strain of CDV HA coupled to the Ftn form are known as *YaH3F, YaH4F,* and *YaH5F*, respectively^39^. BALB/C mice were immunised subcutaneously with each of the three protein-coupled forms and nucleic acids (mRNA), and serum IgG antibodies increased significantly after 28 days, with higher antibody titres in the protein group than in the mRNA group, even maintaining higher antibody titres after 42 days.

The above-mentioned studies show that Ftn nanovaccines can stimulate both B-cell and T-cell activation, thus inducing a complete immune response. Besides, Ftn itself can be used not only as a non-viral vaccine transport carrier, but exhibits also a good effect in enhancing the immunity itself. Self-assembled nanoparticles designed by altering the structure of Ftn induce broader and more effective immunity than traditional influenza vaccines. Protocols need to be standardised so that the biological process of production becomes reproducible and Ftn-based therapies become relatively accessible. Because most self-assembled sequences are derived from species other than humans, one problem with self-assembled protein nanoparticles as vaccines is that off-target immune responses^40^. If they occur, they may limit, to a greater extent, the effectiveness of subsequent booster immunizations or other vaccinations using vaccines containing the same self-assembled structural domains. The mechanism by which Ftn nanovaccines induce a high level and sustained immune response was found to be deposition of Ftns in the lymph nodes of immunised mice and stimulation of specific B lymphocyte maturation; however, this phenomenon was not found in immunised macaques^41^. Thus the specific mechanism of sustained immune response to recombinant Ftn vaccines needs to be further investigated.

## Ferritins as artificial nanozymes

**Figure 3.**
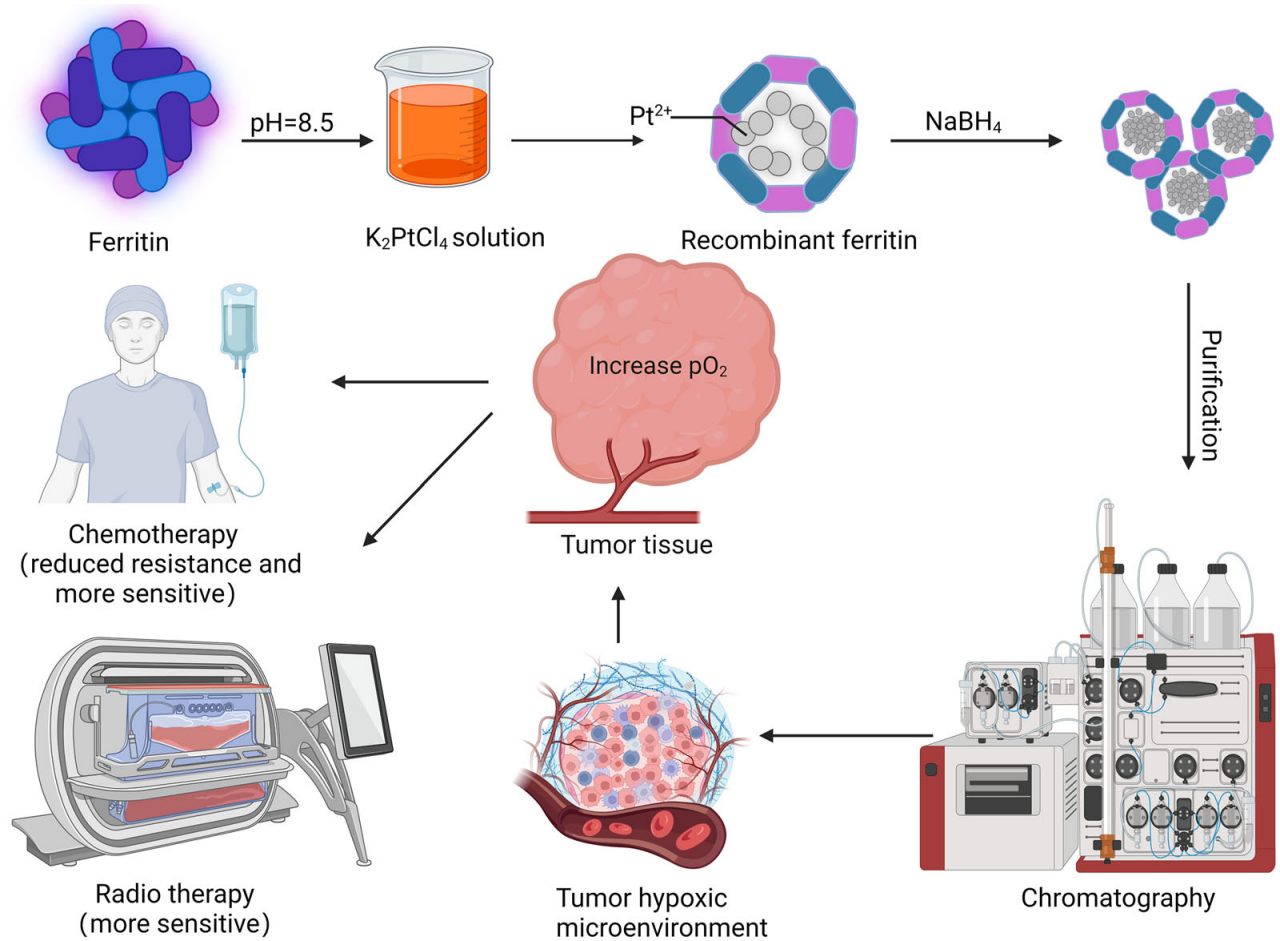
Pt-ferritin develops peroxidase activity to improve the tumour hypoxic microenvironment pO_2_, thus making it more sensitive to chemotherapy and radiotherapy.

Nanozymes are a class of nanomaterials that exhibit intrinsic enzymatic properties and possess enzymatic catalytic properties at the nanoscale that only natural enzymes have^42^. Under mild conditions, artificial nanozyme has certain reaction kinetics^43^. Ftn is a natural nanozyme, which shows intrinsic enzyme-like activities (iron oxide enzyme, peroxidase) and first to report artificially prepared ferromagnetic nanoparticles with peroxidase-like activity^44^. Biomimetic synthesis of nanozymes in Ftn shell and full use of self-assembled nanocage structure can effectively combine the advantages of self-assembled Ftn nanocage and nanozyme. In tumour therapy and chemotherapy, hypoxia has always been the key factor affecting drug resistance. Therefore, reducing O_2_ consumption of tumour mass is one of the ways to alleviate tumour hypoxia^45^, which can increase pO2 in cancerous tumour tissues, improve the hypoxic microenvironment of tumours, restore the sensitivity of tumour cells to chemotherapy and radiotherapy, and thus generally improve the therapeutic effect. What’s more, Ftn-loaded enzymes also have good stability, remaining biologically active after high temperature and detergent treatment. Thus, the peroxidase activity of Ftn could have a promising applicability in tumour therapy (Figure 3).

### Ferrtin is used as a carrier for nanozymes

Transferrin is a receptor on the surface of the cell membrane, which can specifically recognise Ftn^46^. *CD71* is the most common transferrin receptor, which has been shown to allow encapsulation of an artificial transfer hydrogenase (ATHase) based on streptavidin-biotin technology which catalyses the reduction of cycloimine detoxification after the artificial transaminase has also been encapsulated in the cavity of horse spleen apoFtn (HSAF) ^47^. HSAF provides a protective protein shell for precious metal-based artificial enzymes to defend against and adapt to complex physiological environments and deliver them to targeted sites in the body. There are also some advantages in transporting other substances through the modification of Ftn.

In addition to spontaneous formation of iron oxide core nanoparticles, the Ftn cavity can also accommodate a diverse range of artificially prepared metal nanoparticles. Synthesis of platinum nanoparticles in the cavity of desferrin *in vitro*, protein-platinum nanoparticles showed good catalytic efficiency and long-term stability. Subsequently, after the Ftn-receptor-mediated uptake into human intestinal Caco-2 cells, the catalytic activity of the particles was tested. Under externally induced pressure, intracellular concentration of hydrogen peroxide decreases, and cell viability increases^48^. Materials with four enzyme-like activities responsible for reactive oxygen species regulation (oxidase, peroxidase, catalase, and superoxide dismutase) developed with nitrogen-doped porous carbon nanospheres were introduced into Ftn into the lysosome and promoted the production of reactive oxygen species in a tumour-specific manner, leading to significant tumour regression in a mouse model^21^. Therefore, Ftn nanozymes are powerful tools capable of regulating intracellular reactive oxygen species. Loading into Ftns is hence a promising strategy and nanozymes targeting tumour cells can be constructed not only for *in vivo* anticancer applications but also for other biomedicinal purposes such as antiatherosclerotic therapy^49^. Because active macrophages infiltrated by plaques drive atherosclerosis, plaques are more prone to breakage and show a dominant correlation, so HFtn-Fe_3_O_4_ nanozyme can specifically stain unstable ruptured plaques. AgNP obtained in pfFtn is used in areas such as *in vivo* diagnostics, antimicrobial or oncological therapy^50^.

### Ferrtin nanozymes are resistant to high temperature

**Figure 4.**
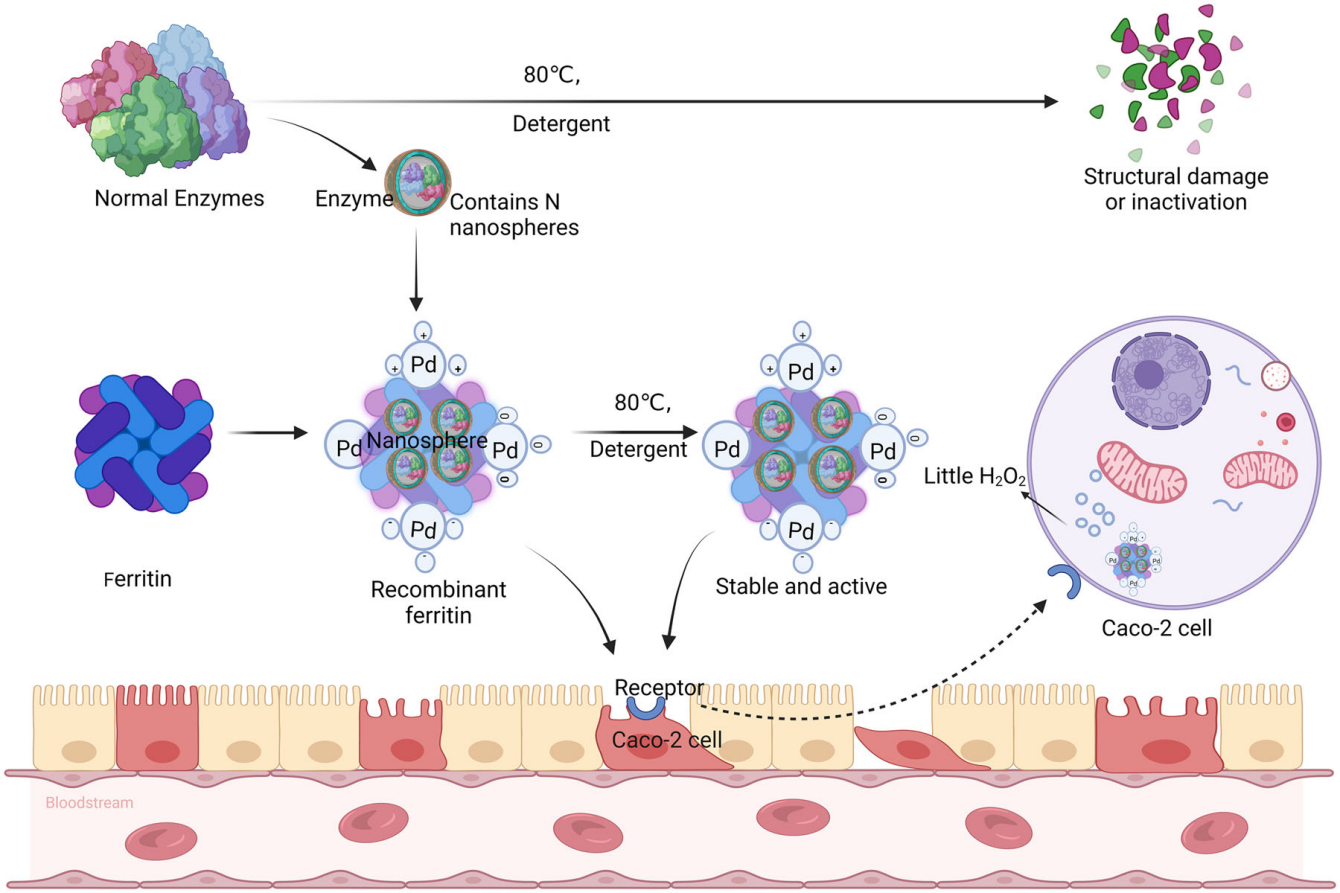
Thermal stability of recombinant ferritin nanozymes.

Furthermore, palladium and silver nanoparticles (NPs) anchored on the outer surface of Ftn form stable suspensions of uncoated particles with multiple catalytic and mimetic enzymatic activities. The nature of pfFtn-Pd Ftn is relatively stable, detergent and an optimum temperature of about 80 °C, high temperature will not destroy the enzyme activity, silver nanoparticles were detected in the presence of sodium dodecyl sulphate (SDS) before mimicking enzyme peroxidase activity, and under this condition, most of the natural enzymes were denatured quickly, while pfFtn-Pd Ftn nanozymes still maintained high stability and catalytic activity due to the excellent thermal stability of pfFtn, showing strong adaptability^51^ (Figure 4).

Ftn nanozymes are an ideal strategy to protect the catalytic activity of artificial metallic nanoparticles from harsh and complex environments, allowing for the delivery of artificial metalloenzymes in living organisms. Artificial nanozymes have the advantages of high biocompatibility, low reaction toxicity, controllable synthesis, and targeted modification, but the disadvantages are that their types are limited. Technology of preparation of nanozymes is still immature, and they are rarely working in living organisms. However, nanozymes field is rapidly advancing and at present, Ftn nanozymes have been successfully tested in certain applications in the medical field. The appearance of Ftn nanozymes also inspires us to learn from natural nanostructures (such as R-chymotrypsin, β-Gal, β-lactamase) to optimise or design nanozyme reasonably.

## Ferritin as bioengineered nanocarrier

Nanocages are good transport materials after Ftn removal of the iron core. Anticancer drugs need to be delivered into tissues and cells with a high level of selectivity and fidelity. Chemotherapy is the main treatment for cancer and despite killing cancer cells; the high levels of the drug can affect the entire network of cells in the body and produce serious side effects^52^. Hence, in the process of delivery, we should not only consider their biocompatibility, toxicity, immunogenicity, and therapeutic index, but also comprehensively consider the disadvantages of the developed nanocarriers, such as non-specific distribution, rapid clearance, uncontrolled drug release, and low bioavailability^53^. However, Ftns can be used specifically to deliver chemotherapeutic drugs and is effective in circumventing these risks. The advantages of the Ftns is the ease of surface modification, high loading capacity, and high biocompatibility making Ftn a multifunctional nanocarrier for the research and development of targeted drugs carrier^54^. Ftn is highly expressed in tumour-associated macrophages, and its down-regulation can destroy the supportive tumour microenvironment, kill cancer cells and increase the sensitivity to chemotherapy^55^. Besides, human heavy chain Ftn (HFtn) can be specifically recognised and internalised by the *CD71*^56^, which is highly expressed in most tumour cells, thus facilitating effective drug-loaded Ftn cellular uptake^22–23^. Ftn has made significant progress in drug encapsulation techniques, starting from single drug encapsulation to two-drug mixed encapsulation and now regional encapsulation, which is highly appealing^57^.

### Recombinant ferritin loads with a single substance to treat various diseases

**Figure 5.**
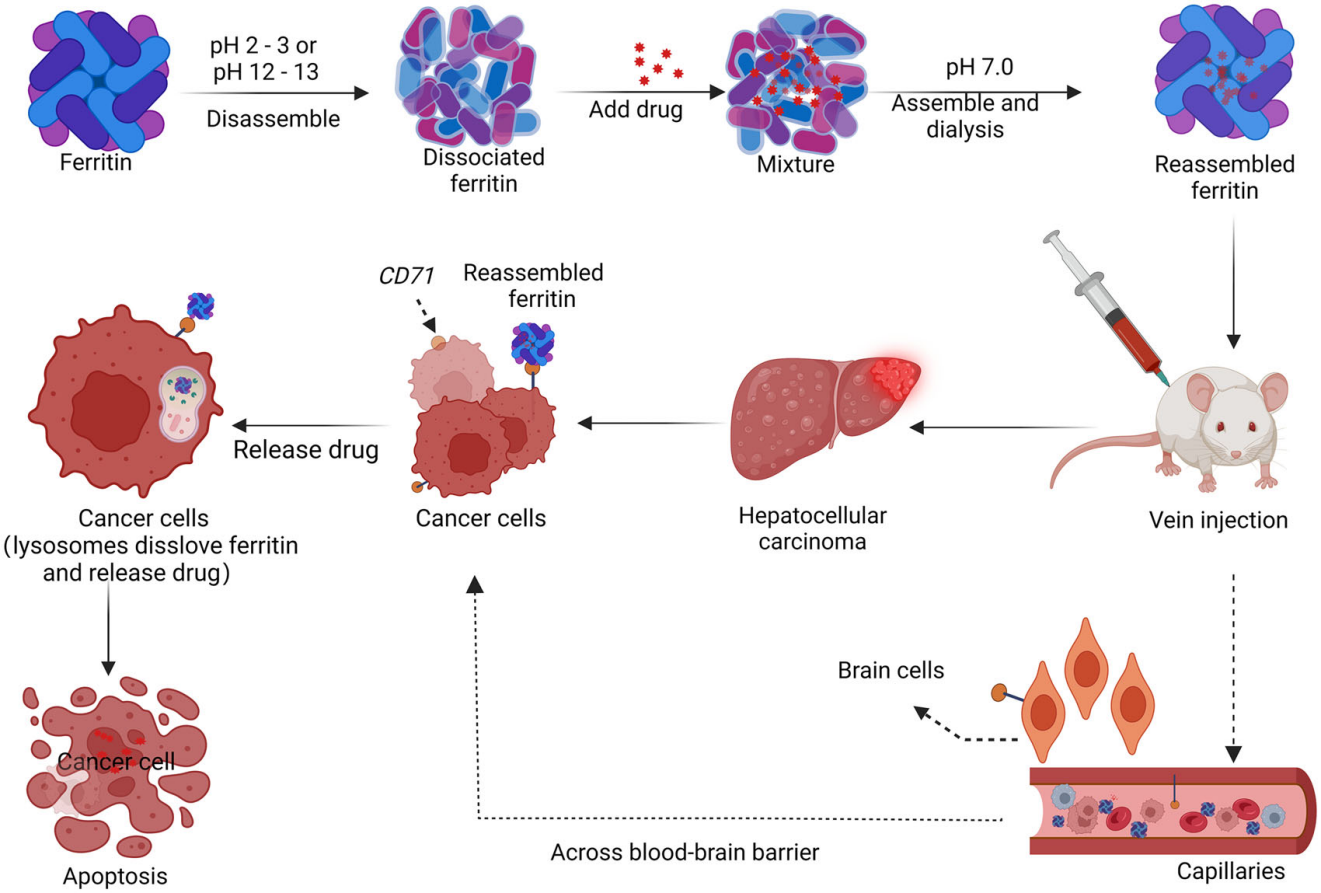
Targeted inductions of cancer cell apoptosis by recombinant ferritin.

Hepatocellular carcinoma (Hcc) is one of the main causes of cancer-related deaths, accounting for the second most common in the world^58^. GRP78 was identified as the membrane receptor of Hcc-targeted peptide SP94^59^, which was displayed on the surface of *Pyrococcus furiosus* Ftn^20^. GRP78-targeted Ftn nanocaged can carry ultra-high dose of Dox for Hcc therapy. Dox encapsulated in HccFtn-Dox was selectively delivered to Hcc cells and effectively killed subcutaneous and pulmonary metastatic Hcc at more than 10-fold higher average levels than those reported for Ftn-based nanocarriers without the GRP78 targeting peptide^20^^,^^60^ (Figure 5). In addition, HccFtn-Dox reduces drug exposure and damage to the liver and kidney, which are the main organs of drug metabolism^21^.

*CD71*, a transferrin receptor that specifically recognises Ftn, was utilised to deliver Ftn-encapsulated Cyt C to an acute promyelocytic leukaemia (APL) NB4 cell line, resulting in apoptosis^46^^,^^61^. This trial was one of the pioneering studies to employ Ftn assembly for Cyt C transportation. In a subsequent *in vivo* study, the surface of the human HFtn nanocage was further functionalised with polyethylene glycol (PEG), which facilitated effective penetration of the mucus barrier and tumour tissue^18^. Using CaP wrapping can effectively shield and protect Ftn, when transported to the weak acid environment of the tumour, and thus release the drug after binding to *CD71* into cancer cells, promoting tumour calcification and immune response^62^.

**Figure 6.**
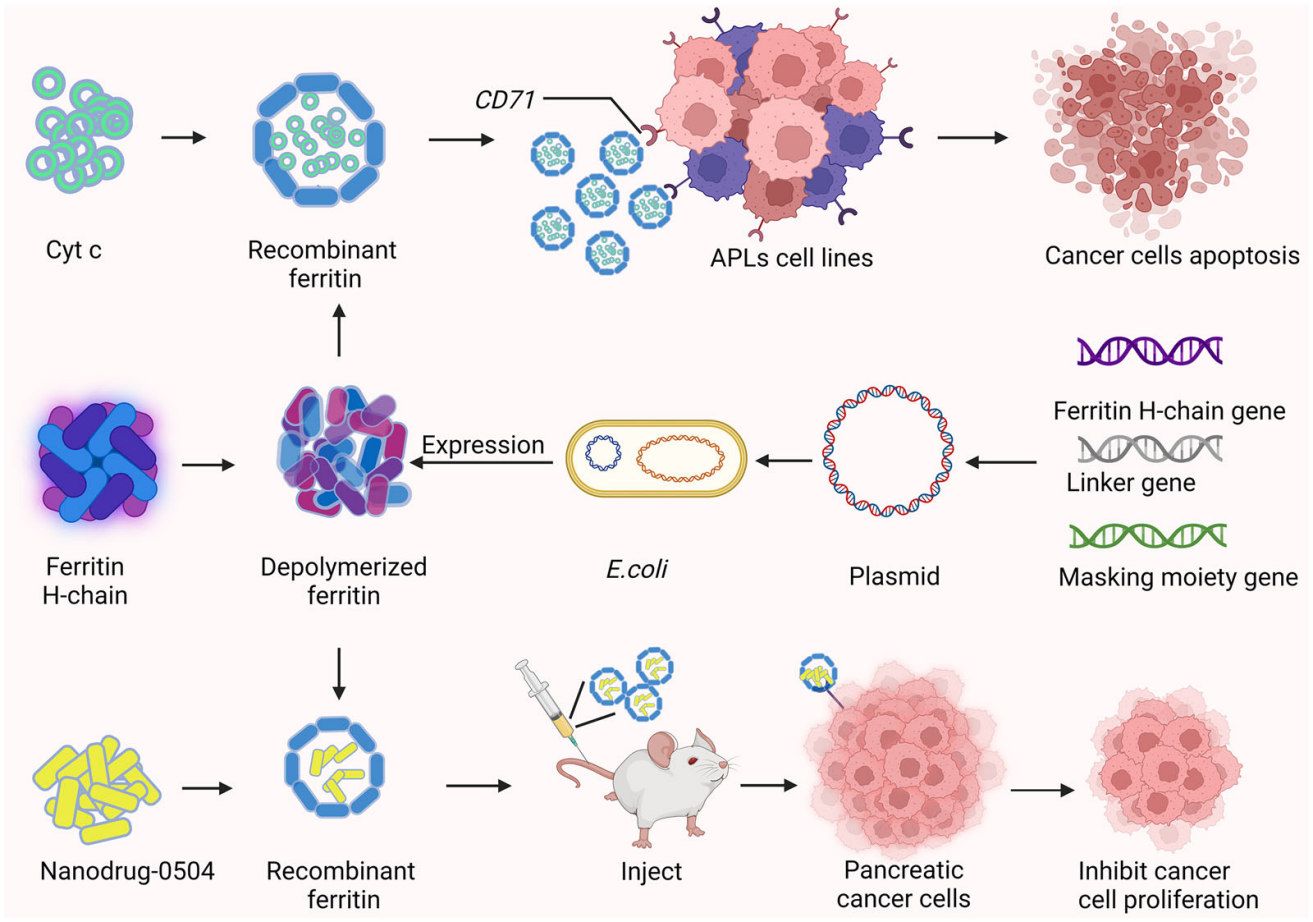
Recombinant ferritin delivery of Cyt c induces in APLs cell apoptosis and nanodrug-0504 inhibits proliferation of pancreatic cancer cells.

In addition, human HFtn can encapsulate different types of drugs within its lumen, can bind to the receptor *CD71*, and the novel nanodrug-0504 contains about 80 molecules of a potent, broad-spectrum, non-camptothecin topoisomerase I inhibitor (Genz-644282) and can be produced industrially in a pure and homogeneously formulated product which can belyophilized^63^. Encapsulation of Genz-644282 with HFtn showed excellent therapeutic efficacy in an *in vivo* pancreatic cancer model (Figure 6), significantly increasing the overall survival rate of the animals^64^.

What’s more, paclitaxel (PTX) is a chemotherapeutic agent, limited in the use by its lack of targeting and insolubility in water^65^. Natural human HFtn nanocages can be used as a carrier for PTX to form HFtn-PTX for targeted delivery, which can bind tumour cells by interacting with *CD71*, leading to endocytosis, and apoptosis assays show that HFtn-PTX has similar apoptotic characteristics to free PTX for MDA-MB-231 cells^66^. The fusion of the penetrating peptide tLyP-1 with the N-terminal end of human HFtn and wraps more PTX molecules in HFtn by endocytosis to form tLyP-1-HFtn-PTX. tLyP-1-HFtn-PTX exhibits greater cytotoxicity and anti-invasive ability, good permeability, and growth inhibition against SMMC-7721 spheroids. Animal experiments on BABL/c nude mice focussed on selective accumulation and penetration into the tumour mass *in vivo* showed higher therapeutic efficacy and lower systemic toxicity^67^.

HFtn can be targeted by recognising *CD71*, absorbed through endocytosis and then degraded by lysosomes, and epitope-modified HFtn can further show better efficacy and potential^46^. Curcumin loading can be used in the same way, with curcumin encapsulated in HFtn to make CHFtn, which inhibits both MDA-MB-468 and MDA-MB-231 cell lines of triple-negative breast cancer (TNBC), suppressing both at G2/M and G0/G1 stages, respectively, and can be used in TNBC treatment^68^. Similarly, the increased cellular uptake and anticancer activity of geftinib-loaded HFtn was found in breast cancer (SKBR3) cells. Compared to HFtn alone, HFtn loaded with gefitinib exhibits sustained pH-responsive release of gefitinib, allowing effective delivery of gefitinib to breast cancer cells^69^.

Till now, more than a dozen drug molecules were successfully introduced into the Ftn nanocages, indicating that the one-step method is a powerful vehicle for the construction of drug-loaded Ftn delivery systems^70^. Human HFtn has a natural two-fold axis loading channel with an average diameter of 0.9 nm^71^. In addition, the open state of this drug entry channel is sensitive to temperature changes between 4 °C-37 °C, the natural drug channel is closed when incubated at 37 °C-65 °C, the amount of Dox loaded into HFtn gradually increases and reaches the peak at 65 °C, and can stably encapsulate about 90 Dox molecules^71^. Temperature directly determines the opening and closing of such channels^72^, as 30 mM urea can expand the Ftn native channel size^73^. Loading Dox through channel into HFn nanocages allows for higher drug loading efficiency, higher HFtn recovery, and better stability^71^. Moreover, channel-loaded HFn-Dox has excellent biosafety and significantly improved antitumor activity^71^. For this purpose, the mixture was incubated at the appropriate pH to obtain complexes of Ftn and the target drug. The electrostatic potential facilitated the passage of drug molecules through the pores, across the Ftn shell, and the deposition of drug within the Ftn cavity. The constructed Dox-HFtn loading was more than 3 times higher than the free dosage, and the recovery of Dox was improved by 10 times^74^.

### Ferritin as a carrier of photothermal and photosensitizers for cancer therapy

In recent years, Ftn has been shown to play an important role in phototherapy as a carrier of photothermal agent and photosensitizer and can realise the combined treatment of chemotherapy and phototherapy^75^. Photothermal therapy (PTT) with Ftn cages is a new direction in targeted therapy, such as encapsulation of copper Ftn followed by laser irradiation, which kills 100% of human glioblastoma cells and without obvious toxic side effects^76^. Prussian blue (PB)-modified Ftn nanoparticles (PB-Ft NPs) have peroxidase activity, which can catalyse the oxidation of chemotherapy drug 3,3′,5,5′-tetramethylbenzidine by hydrogen peroxide into oxidised TMB, and can successfully inhibit the growth of murine breast cancer cell line (4T1)^77^. Ftn modified by CGKRK polypeptide was constructed by genetic engineering. Fluorescence intensity test confirmed that targeting CGKRK peptides modified Ftn maintained ROS-producing ability. Photodynamic therapy (PDT) is a widely known oncology therapy that can be combined with PTT to treat tumours. In the PTT + PDT group, the tumour subsided, and the tumour did not recur after 16 days of treatment, indicating the advantages of synergistic treatment^78^. PB-Ft nanoparticles are a good sensitiser for tumour chemotherapy. PTT mediated by PB-Ft NPs may have the ability to reverse drug resistance. Folic acid was coupled to the surface of Ftn as a tumour-targeting ligand, then Ftn nanocages were subsequently loaded onto ZnF16Pc. The resulting recombinant Ftn nanoconjugates could effectively inhibit 4T1 tumours *in vivo* and effectively inhibit tumour growth and tumour metastasis in mice under light exposure^79^.

### Ferritin delivers nucleic acid-based drugs

The use of recombinant Ftn for polypeptide and gene delivery can also regulate molecular release. Nucleic acid-based drugs, including small interfering RNAs (siRNAs), are becoming increasingly important due to their remarkable efficiency in the treatment of various diseases^80^. The siRNA can be encapsulated in unmodified human Ftn. The encapsulation of siRNA into the Ftn is not dependent on the sequence of the siRNA, and the siRNA is protected from degradation, resulting in efficient delivery into human tumorigenic cells, human primary mesenchymal stem cells, and peripheral blood mononuclear cells^81^. At the siRNA concentration of 10 nM, the high-efficiency silencing of the target gene can be achieved by using Ftn in tumour cells and PBMCs as a delivery agent, immune activation of human T cells and B cells is not induced, and the internal anti-inflammatory effect is displayed, indicating that human Ftn can be used for siRNA delivery under the condition of infection or inflammatory disease.

The choice of phosphorylated Ftn nanocage avoids these risks and has a high drug encapsulation rate^82^. What’s more, in the simulated gastrointestinal tract test, Ftn can slow down the release rate of procyanidins (PCS)^83^. In addition, Ftn has been also used to improve water solubility, stability, and cell uptake efficiency of rutin and epigallocatechin gallate^84^.

### Ferritin nanocarriers can carry two drugs simultaneously

The fusion of the C-end of the human HFtn subunit with an optimised hydrophobic peptide resulted in a modification of the inner surface of the Ftn cavity^85^. This modified cavity was able to simultaneously load Dox and striline, leading to significant anti-tumour effects in animal models of neuroglioma, metastatic liver cancer, and drug-resistant breast cancer^85^. Curcumin and quercetin have been shown to have anti-tumour properties by blocking different signalling pathways^86^. These two bioactive substances are co-encapsulated within Ftn, which enhances their synergistic cytotoxicity by increasing the loading rate and targeting cancer cells^86^. Shrimp Ftn can separate chlorogenic acid and β-carotene through reassembly and emulsification^87^. In addition to its potential in treating common tumour diseases, ATP-loaded Ftn has also shown promising in treating asthenospermia by crossing the blood-testis barrier^88^. Therefore, the use of Ftn encapsulation can reduce drug clearance, extend the half-life, reduce drug dosage or liver toxicity. Ftn can serve as a promising drug or nutrient carrier for delivery *in vivo*.

## Ferritin as bioimaging tool

**Figure 7.**
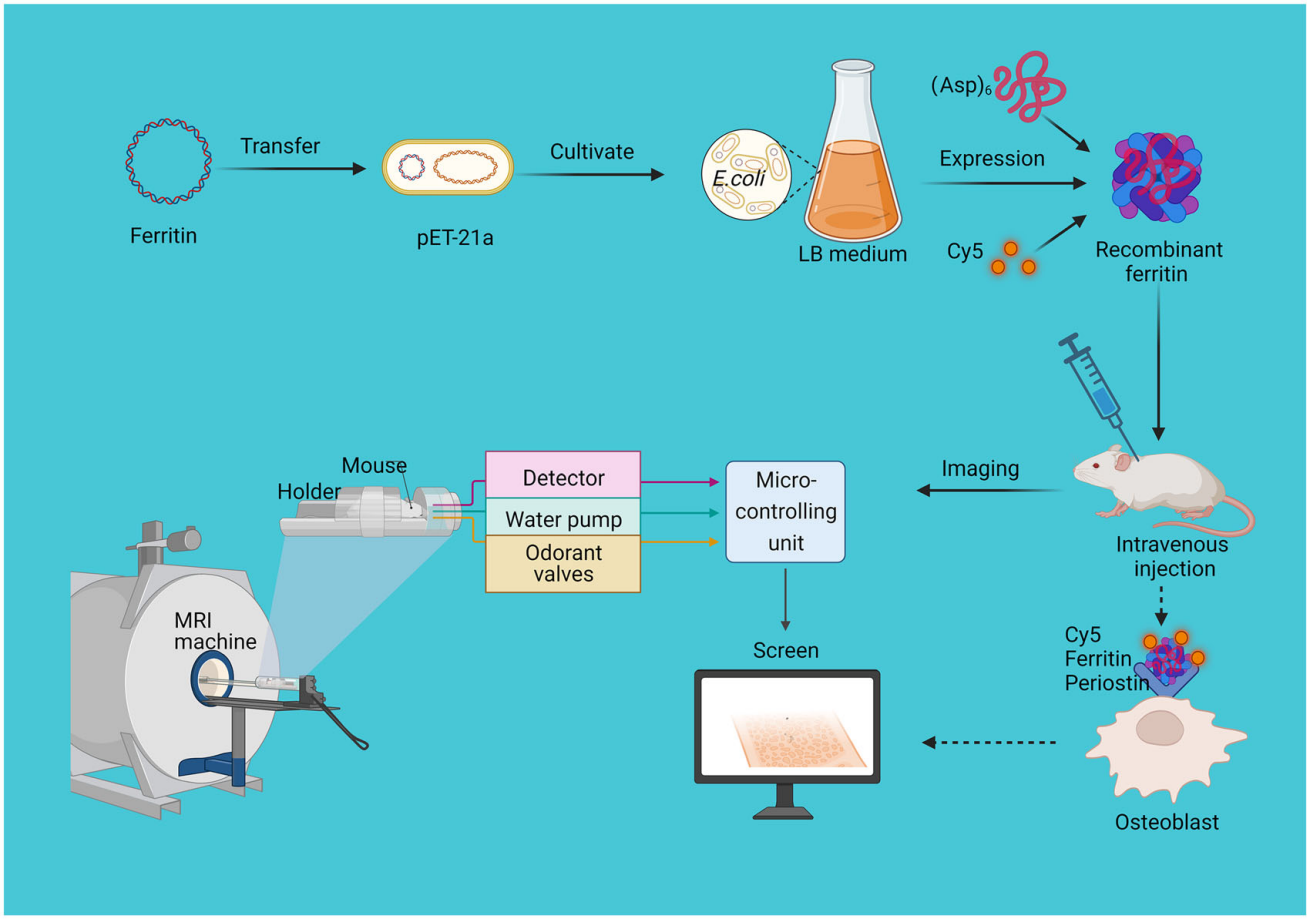
Recombinant ferritin in bone imaging.

A bone-targeting peptide that specifically binds osteoblasts and hydroxyapatite was fused to the N-terminal part of the Human HFtn, and the hybrid Ftn nanoparticles were labelled with fluorescent dyes. Upon binding of the modified Ftn to osteoblasts and hydroxyapatite specific fluorescent signals were observed in the lower limbs, indicating that recombinant Ftn nanoparticles could specifically recognise the bone tissue, and in the future, Ftn could be applied in clinical treatment of bone diseases^89^ (Figure 7). Bifunctional carbon dots (CDs) attached to the surface of HFtn interacting with Dox emitting bright red fluorescence can be used for sensitive *in vivo* bioimaging^90^. Despite the potential of iron oxide nanoparticles (SPIO) as a blood contrast agent, medical applications remain limited due to their short half-life of a few hours. The contrast agent prepared by temporarily opening the pores of the red blood cell membrane and encapsulating SPIO nanoparticles in red blood cells (RBCs) has a long half-life, which can be used as a magnetic resonance imaging (MRI) contrast agent. Due to the short half-life of standard superparamagnetic iron oxide (SPIO) *in vivo*^91^, at 2.5 mg Fe/kg, standard SPIO is only stable for 7 days for MRI bioimaging of the liver in mice, whereas deferrin iron in 45 days still can be detected^92^. Terahertz (THz) bioimaging has attracted much attention due to its ability to acquire physicochemical information in a label-free, non-invasive and non-ionizing manner. To date, a variety of macroscopic biological samples (such as burned skin, brain tissue, and various cancerous tissues) have been imaged using THz imaging, with graphene chosen as the substrate for terahertz presentation^93^. Ftn can be used for disease diagnosis and provide a non-invasive and highly sensitive imaging method. By graphene-mediated of THz scattering type scanning near-field optical microscopy, Ftn and IgG can direct imaging^94^. The THz direct imaging of individual biomolecules using Ftn opens up a new avenue for imaging individual biomolecules by coupling effects to real-time monitoring and does not require repeat injections.

Besides, bone marrow mesenchymal stromal cells (BMSCs) were transduced with a lentivirus containing a shuttle plasmid carrying the Ftn heavy chain 1 (Fth1) gene, and the migration and proliferation of mesenchymal cells in a rat stroke model were monitored long-term using magnetic resonance imaging (MRI) of Ftn transgene expression^95^. Ftn expression in stromal cells was evaluated with western blotting and immunofluorescent staining. Bone marrow mesenchymal stromal cells (BMSCs) were transduced with a lentivirus containing a shuttle plasmid (pCDH-CMV-MCS-EF1-copGFP) carrying the HFtn gene. After middle cerebral artery occlusion surgery, Fth1-BMSCs and superparamagnetic iron oxide (SPIO)-labelled BMSCs were injected intravenously into the internal jugular, and monitoring imaging and signal intensity by observing *in vitro* and *in vivo* revealed that MRI signal intensity of SPIO-BMSCs gradually decreased over time and that Prussian blue-stained cells were present around and in the centre of the infarct area in both transplantation models. Fth1-BMSCs transplanted for the treatment of focal cerebral infarction was safe, reliable, and traceable by MRI. Fth1 marker was more stable than the SPIO marker and suitable for long-term follow-up.

The application of recombinant Ftn is not only in disease diagnosis, but also in the detection of harmful substances. Methylmercury (MeHg^+^) accumulates mainly in the brain and therefore *in vivo* imaging detection of MeHg^+^ in the brain is crucial^96^. By integrating the bioimaging of gold nanoclusters (Au NCs), the fluorescence of Au NCs quenched by MeHg^+^, and the brain-targeting function of the constructed 16-mer shell-like Ftn (7 A), the prepared 7 A-Au NCs were found to be not only suitable for bioimaging of BBB endothelial cells, but also an effective probe for brain-specific bioimaging MeHg^+^ detection. The recombinant Ftn has the advantages of low toxicity, high sensitivity, and long-term presence in the human body, no need for repeated injections, and easy detection as a bioimaging material. In the future, Ftn will play an important role in the clinical diagnosis of human diseases and detection of harmful substances (Table 1).

**Table 1.** A summary of recombinant ferritin in multimodal nanomedicine.

Ferritin carrier	Expression system	Surface modification	Targeting receptor	Major utilisation	References
H. pylori	HEK293T	RBD and Ftn spaced by a SSGGASVLA linker	S protein	viral vaccine	Kim et al.^13^
Thermococcus furiosus Ftn	–	–	GRP78	targeted therapy for Hcc	Jiang et al.^20^
–	HepG2	Covalent attachment of HFtn to N-PCNSs was performed using 1-ethyl-3-(3-dimethyl aminopropyl) carbodiimide (EDC) and N-hydroxysuccinimide (NHS)	–	artificial enzymes	Fan et al.^21^
H. pylori	HEK293F	S protein and Ftn spaced by an SGG linker	S protein	SARS-CoV-2 vaccine	Powell et al.^24^
Rat H-chain	HEK293F CHO	–	EV71	RSV vaccine	Wang et al.^34^
Human H-chain	E. coli	Loading Fe^2+^ into the cavities of HFtn nanocages through the iron ion channel on the surface of HFtn nanocage	–	artificial enzyme	Wang et al.^49^
Pyrococcus furiosus Ftn	BL21-CodonPlus (DE3)-RIL	–	–	artificial enzymes	Peskova et al.^51^
H. pylori-bullfrog hybrid	HEK293F	HA and Ftn spaced by an SG linker	RBD of HA	viral vaccine	Kalathiya et al.^37^
H. pylori	HEK-293T	Ftn-coupled HA sequences	YaH3F, YaH4F, and YaH5F	CDV vaccine	Wang et al.^39^
–	Drosophila S2 cell	Trimeric SARS-CoV-2 S protein devoid of transmembrane domain	S protein	viral vaccine	Lai et al.^25^
H. pylori bullfrog Ftn	HEK293F	gp350 and Ftn spaced by an (SG3)_2_ linker	gp350	EBV vaccine	Kanekiyo et al.^27^
H. pylori	HEK293F	CD5 leader sequence and a SGG spacer were fused to the gene fragment encoding Ftn	HA	H1N1 vaccine	Kanekiyo et al.^28^
T. ni L- and H-chains	HEK293F	gp140 and Ftn spaced by a (GS)_5_ linker	gp140	HIV-1 vaccine	Georgiev et al.^29^
H. pylori bullfrog Ftn	BL21	N-terminal fusion of alpha-hemolysin with Ftn CDS	alpha-hemolysin	Staphylococcus aureus vaccine	Wei et al.^31^
Rat H-chain	BL21(DE3)	SpyCatcher fused to C-terminal of preS1; SpyTag and Ftn spaced by a (G4S)_3_ linker	preS1 of HBV	HBV vaccine	Wang et al.^32^
H. pylori (N19Q)^2^	–	–	TLR4/NF-κB	DC-based vaccine	Qu et al.^33^
Human HFn (Prussian blue PB-modifified)	–	Recombination of encapsulated PB after Ftn depolymerisation	–	breast cancer targeted therapy	Li et al.^77^
Human HFn	–	–	TfR1	breast cancer targeted therapy	Li et al.^66^
Human H-chain	–	tLyP-1 was fused to the N-terminal end of human HFtn, encapsulating the PTX molecule into HFtn to form tLyP-1-HFtn-PTX	TfR1	breast cancer targeted therapy	Ma et al.^67^
Human H-chain	–	HFtn - Curcumin	–	TNBC therapy	Pandolfi et al.^68^
humanized archaeal Ftn (HumFt)	*E*. Coli	–	–	deliver cyt C	Macone et al.^61^
Human H-chain	–	folic acid	folic acid receptor	deliver folic acid	Zhen et al.^79^
Human HFtn	–	–	–	deliver genz-644282	Falvo et al.^64^
Human Ftn	–	siRNA	PBMCs	genetic silence	Li et al.^81^
–	BL21 (DE3)	Osteoblast and hydroxyapatite binding to HFtn by a GSS linker	OB-Ftn and HA-Ftn	bone disease imaging	Kim et al.^89^
Human HFn (CDs modifified)	–	–	–	beast cancer imaging	Yao et al.^90^
–	HEK-293T	–	–	cerebral infarct imaging	Huang et al.^95^
Ftn-like protein, 7 A,	–	Encapsulated Au- NCs	–	methylmercury imaging	Lv et al.^96^

## Concluding remarks and perspectives

Based on the suitable immunogenicity of recombinant Ftn, various recombinant protein vaccines have been developed by fusing genes of different pathogens including HIV, SARS-CoV-2, and influenza. Ftn nanozymes show promise for the treatment of cancer, atherosclerosis, and diagnostic. The advantages of Ftn nanozymes are stability, controlled size synthesis, and the ability to be further modified. As targeted cancer drugs, Ftn delivers PTX to mammary cancer tissues, and also reshapes the tumour immunosuppressive microenvironment, and activates intrinsic and adaptive immunity, leading to durable immune memory and anti-tumour effects. In addition, Ftn has been developed as a drug delivery vehicle for hundreds of drug delivery systems. Recombinant Ftn is a bioimaging material that persists in the body and can be used for liver diagnostics, brain infarction detection, and the detection of MeHg^+^.

Ftn nanoparticles are biocompatible with almost all living organisms, the raw materials are cheap and easy to obtain, the preparation process is economical and environmentally friendly, the structure of the product is well defined, the chemical properties are relatively stable and it is easy to degrade and metabolise *in vivo*. In addition, *in vitro* recombinant Ftn is simple to synthesise, easy to modify, and easy to implement. These advantages make it very useful for clinical applications. Due to the unique autoloading ability, numerous different types of molecules can be successfully loaded onto Ftn, especially for the loading and targeting of a wide range of active substances, showing its great potential as a nanocarrier in the fields of disease diagnosis, drug delivery, and bioimaging. Currently, nanomaterials are widely used in medicine, pharmacy, and bioassays. However, there are still some issues concerning the development of Ftn nanoparticles that need to be addressed. for example, the immunogenicity of human-derived Ftn and heterologous Ftn vaccines in different mammals or primates are not fully explored and need to be studied. As carriers for loading drugs, Ftn subunits with different loading ranges for target proteins or peptide chain sizes should be studied. The metabolic pathways of heterologous Ftn nanocages or their derivatives *in vivo* are not still clear. The encapsulation rate and drug loading capacity of Ftn for different drugs should be tested. There are still no systematic studies on the changes in various properties of Ftn after modification, so the safety of Ftn surface modification is open to debate and observation. The modification of the contact surfaces between Ftn subunits controls self-assembly, but self-assembly at the three-dimensional level still needs to be studied in depth. The self-assembled structures of different subunits allow for reactions with a certain degree of stochasticity and uncertainty, as well as the influence of spatial site resistance effects, which require precise control of the reaction conditions to obtain more satisfactory results. In addition, Ftn nanomaterials still present some problems *in vivo*, mainly including oxidative stress, inflammation, and genotoxicity generated by iron ions, which at the cellular level can cause the malfunction of major organelles such as autophagosomes, lysosomes, endoplasmic reticulum, and mitochondria. Therefore, due to the complex design of Ftn nanoparticles, it is difficult to meet the criteria for targeted drug delivery and clinical applications still need time.

The development prospect of recombinant Ftn nanoparticles is to expand their physiological functions through surface-modified materials while ensuring their high biocompatibility and biodegradability, in order to develop more products adapted to clinical conditions. It is important to note that the Ftn modification strategy to be used depends primarily on the specific function and the specific goal that the modification gives to the Ftn, rather than simply pursuing its multifunctionalisation. The combination of biological and chemical modification of Ftn surfaces allows for site-specific modifications and the attachment of various macromolecules, including affinity markers, antibodies, fluorescent, glycans, nucleic acids, and target peptides, giving Ftn new properties and significantly broadening its range of applications. Therefore, combining chemical and biological modifications to complement each other’s strengths and weaknesses to achieve the modification and multifunctional modification of Ftn is one of the important directions for future development. It is believed that as the research progresses and technology advances, more recombinant Ftn nanoparticles with more diversified structural composition, more intelligent and comprehensive functions will be used in clinical medical research, and Ftn will have more widespread biomedical applications and show its good application prospects.

## References

[1] Zhang N, Yu XQ, Xie JX, Xu HM. New insights into the role of ferritin in iron homeostasis and neurodegenerative diseases. Mol Neurobiol. 2021;58(6):2812–2823. 33507490 10.1007/s12035-020-02277-7

[2] Zang JC, Chen H, Zhao GH, Wang FD, Ren FZ. Ferritin cage for encapsulation and delivery of bioactive nutrients: from structure, property to applications. Crit Rev Food Sci Nutr. 2017;57(17):3673–3683. 26980693 10.1080/10408398.2016.1149690

[3] Adameyko KI, Burakov AV, Finoshin AD, Mikhailov KV, Kravchuk OI, Kozlova OS, Gornostaev NG, Cherkasov AV, Erokhov PA, Indeykina MI, et al. Conservative and atypical ferritins of sponges. IJMS. 2021;22(16):8635. 34445356 10.3390/ijms22168635 PMC8395497

[4] Zhen ZP, Tang W, Todd T, Xie J. Ferritins as nanoplatforms for imaging and drug delivery. Expert Opin Drug Deliv. 2014;11(12):1913–1922. 25070839 10.1517/17425247.2014.941354 PMC4885637

[5] Garcia-Casal MN, Pasricha SR, Martinez RX, Lopez-Perez L, Pena-Rosas JP, Cochrane Tobacco Addiction Group. Serum or plasma ferritin concentration as an index of iron deficiency and overload. Cochrane Database Syst Rev. 2021;2021(5):CD011817. 10.1002/14651858.CD011817.pub2 PMC8142307 34028001

[6] Ueda N, Takasawa K. Impact of inflammation on ferritin, hepcidin and the management of iron deficiency anemia in chronic kidney disease. Nutrients. 2018;10(9):1173. 30150549 10.3390/nu10091173 PMC6163440

[7] Mahroum N, Alghory A, Kiyak Z, Alwani A, Seida R, Alrais M, Shoenfeld Y. Ferritin-from iron, through inflammation and autoimmunity, to COVID-19. Journal of Autoimmunity. 2022;126:102778. 34883281 10.1016/j.jaut.2021.102778 PMC8647584

[8] Sun Q, Yang F, Wang H, Cui F, Li Y, Li S, Ren Y, Lan W, Li M, Zhu W, et al. Elevated serum ferritin level as a predictor of reduced survival in patients with sporadic amyotrophic lateral sclerosis in China: a retrospective study. Amyotroph Lateral Scler Frontotemporal Degener. 2019;20(3-4):186–191. 30652532 10.1080/21678421.2018.1555599

[9] Gossard TR, Trotti LM, Videnovic A, St Louis EK. Restless legs syndrome: contemporary diagnosis and treatment. Neurotherapeutics. 2021;18(1):140–155. 33880737 10.1007/s13311-021-01019-4 PMC8116476

[10] Devos D, Cabantchik ZI, Moreau C, Danel V, Mahoney-Sanchez L, Bouchaoui H, Gouel F, Rolland A-S, Duce JA, Devedjian J-C, et al. Conservative iron chelation for neurodegenerative diseases such as Parkinson’s disease and amyotrophic lateral sclerosis. J Neural Transm. 2020;127(2):189–203. 31912279 10.1007/s00702-019-02138-1

[11] Demchuk AM, Patel TR. The biomedical and bioengineering potential of protein nanocompartments. Biotechnol Adv. 2020;41:107547. 32294494 10.1016/j.biotechadv.2020.107547

[12] Rodrigues MQ, Alves PM, Roldao A. Functionalizing ferritin nanoparticles for vaccine development. Pharmaceutics. 2021;13(10):1621. 34683914 10.3390/pharmaceutics13101621 PMC8540537

[13] Kim Y-I, Kim D, Yu K-M, Seo HD, Lee S-A, Casel MAB, Jang S-G, Kim S, Jung WRam, Lai C-J, et al. Development of spike receptor-binding domain nanoparticles as a vaccine candidate against SARS-CoV-2 infection in ferrets. Mbio. 2021;12(2):e00230–21. 33653891 10.1128/mBio.00230-21 PMC8092224

[14] Xu XL, Tian KW, Lou XF, Du YZ. Potential of ferritin-based platforms for tumor immunotherapy. Molecules. 2022;27(9):2716. 35566065 10.3390/molecules27092716 PMC9104857

[15] Shepherd BO, Chang D, Vasan S, Ake J, Modjarrad K. HIV and SARS-CoV-2: tracing a path of vaccine research and development. Curr HIV/AIDS Rep. 2022;19(1):86–93. 35089535 10.1007/s11904-021-00597-4 PMC8795326

[16] Pollet J, Chen WH, Strych U. Recombinant protein vaccines, a proven approach against coronavirus pandemics. Adv Drug Deliv Rev. 2021;170:71–82. 33421475 10.1016/j.addr.2021.01.001 PMC7788321

[17] Palombarini F, Di Fabio E, Boffi A, Macone A, Bonamore A. Ferritin nanocages for protein delivery to tumor cells. Molecules. 2020;25(4):825. 32070033 10.3390/molecules25040825 PMC7070480

[18] Huang X, Chisholm J, Zhuang J, Xiao Y, Duncan G, Chen X, Suk JS, Hanes J. Protein nanocages that penetrate airway mucus and tumor tissue. Proc Natl Acad Sci USA. 2017;114(32):E6595–E6602. 28739953 10.1073/pnas.1705407114 PMC5559033

[19] Ghandehari H, Chan H-K, Harashima H, MacKay JA, Minko T, Schenke-Layland K, Shen Y, Vicent MJ. Advanced drug delivery 2020-Parts 1,2 and 3 Preface. Adv Drug Deliv Rev. 2020;156:1–2. 33308449 10.1016/j.addr.2020.11.003

[20] Jiang B, Zhang R, Zhang J, Hou Y, Chen X, Zhou M, Tian X, Hao C, Fan K, Yan X, et al. GRP78-targeted ferritin nanocaged ultra-high dose of doxorubicin for hepatocellular carcinoma therapy. Theranostics. 2019;9(8):2167–2182. 31149036 10.7150/thno.30867 PMC6531302

[21] Fan K, Xi J, Fan L, Wang P, Zhu C, Tang Y, Xu X, Liang M, Jiang B, Yan X, et al. In vivo guiding nitrogen-doped carbon nanozyme for tumor catalytic therapy. Nat Commun. 2018;9(1):1440. 29650959 10.1038/s41467-018-03903-8 PMC5897348

[22] Sun XR, Hong YL, Gong YB, Zheng SS, Xie DH. Bioengineered ferritin nanocarriers for cancer therapy. IJMS. 2021;22(13):7023. 34209892 10.3390/ijms22137023 PMC8268655

[23] Houser KV, Chen GL, Carter C, Crank MC, Nguyen TA, Burgos Florez MC, Berkowitz NM, Mendoza F, Hendel CS, Gordon IJ, et al. Safety and immunogenicity of a ferritin nanoparticle H2 influenza vaccine in healthy adults: a phase 1 trial. Nat Med. 2022;28(2):383–391. 35115706 10.1038/s41591-021-01660-8 PMC10588819

[24] Powell AE, Zhang K, Sanyal M, Tang S, Weidenbacher PA, Li S, Pham TD, Pak JE, Chiu W, Kim PS, et al. A single immunization with spike-functionalized ferritin vaccines elicits neutralizing antibody responses against SARS-CoV-2 in mice. ACS Cent Sci. 2021;7(1):183–199. 33527087 10.1021/acscentsci.0c01405 PMC7805605

[25] Lai C-Y, To A, Wong TAS, et al. Recombinant protein subunit SARS-CoV-2 vaccines formulated with CoVaccine HT adjuvant induce broad, Th1 biased, humoral and cellular immune responses in mice. bioRxiv. 2021 10.1016/j.jvacx.2021.100126 PMC8570651 34778744

[26] Swanson KA, Rainho-Tomko JN, Williams ZP, Lanza L, Peredelchuk M, Kishko M, Pavot V, Alamares-Sapuay J, Adhikarla H, Gupta S, et al. A respiratory syncytial virus (RSV) F protein nanoparticle vaccine focuses antibody responses to a conserved neutralization domain. Sci Immunol. 2020;5(47):eaba6466. 32358170 10.1126/sciimmunol.aba6466

[27] Kanekiyo M, Bu W, Joyce MG, Meng G, Whittle JRR, Baxa U, Yamamoto T, Narpala S, Todd J-P, Rao SS, et al. Rational design of an epstein-barr virus vaccine targeting the receptor-binding site. Cell. 2015;162(5):1090–1100. 26279189 10.1016/j.cell.2015.07.043 PMC4757492

[28] Kanekiyo M, Wei C-J, Yassine HM, McTamney PM, Boyington JC, Whittle JRR, Rao SS, Kong W-P, Wang L, Nabel GJ, et al. Self-assembling influenza nanoparticle vaccines elicit broadly neutralizing H1N1 antibodies. Nature. 2013;499(7456):102–106. 23698367 10.1038/nature12202 PMC8312026

[29] Georgiev IS, Joyce MG, Chen RE, Leung K, McKee K, Druz A, Van Galen JG, Kanekiyo M, Tsybovsky Y, Yang ES, et al. Two-component ferritin nanoparticles for multimerization of diverse trimeric antigens. ACS Infect Dis. 2018;4(5):788–796. 29451984 10.1021/acsinfecdis.7b00192 PMC11103579

[30] von Hoven G, Rivas AJ, Neukirch C, Klein S, Hamm C, Qin Q, Meyenburg M, Füser S, Saftig P, Hellmann N, et al. Dissecting the role of ADAM10 as a mediator of Staphylococcus aureus alpha-toxin action. Biochem J. 2016;473(13):1929–1940. 27147619 10.1042/BCJ20160062

[31] Wei J, Cheng X, Zhang Y, Gao C, Wang Y, Peng Q, Luo P, Yang L, Zou Q, Zeng H, et al. Identification and application of a neutralizing epitope within alpha-hemolysin using human serum antibodies elicited by vaccination. Mol Immunol. 2021;135:45–52. 33873093 10.1016/j.molimm.2021.03.028

[32] Wang W, Zhou X, Bian Y, Wang S, Chai Q, Guo Z, Wang Z, Zhu P, Peng H, Yan X, et al. Dual-targeting nanoparticle vaccine elicits a therapeutic antibody response against chronic hepatitis B. Nat Nanotechnol. 2020;15(5):406–416. 32123380 10.1038/s41565-020-0648-y PMC7223715

[33] Qu Z, Guo Y, Li M, Cao C, Wang J, Gao M. Recombinant ferritin nanoparticles can induce dendritic cell maturation through TLR4/NF-kappaB pathway. Biotechnol Lett. 2020;42(12):2489–2500. 32567013 10.1007/s10529-020-02944-8

[34] Wang Z, Xu L, Yu H, Lv P, Lei Z, Zeng Y, Liu G, Cheng T. Ferritin nanocage-based antigen delivery nanoplatforms: epitope engineering for peptide vaccine design. Biomater Sci. 2019;7(5):1794–1800. 30888360 10.1039/c9bm00098d

[35] Zhang B, Chao CW, Tsybovsky Y, Abiona OM, Hutchinson GB, Moliva JI, Olia AS, Pegu A, Phung E, Stewart-Jones GBE, et al. A platform incorporating trimeric antigens into self-assembling nanoparticles reveals SARS-CoV-2-spike nanoparticles to elicit substantially higher neutralizing responses than spike alone. Sci Rep. 2020;10(1):18149. 33097791 10.1038/s41598-020-74949-2 PMC7584627

[36] Souza PFN, Amaral JL, Bezerra LP, Lopes FES, Freire VN, Oliveira JTA, Freitas CDT. ACE2-derived peptides interact with the RBD domain of SARS-CoV-2 spike glycoprotein, disrupting the interaction with the human ACE2 receptor. J Biomol Struct Dyn. 2022;40(12):5493–5506. 33427102 10.1080/07391102.2020.1871415 PMC7876913

[37] Kalathiya U, Padariya M, Fahraeus R, Chakraborti S, Hupp TR. Multivalent display of SARS-CoV-2 Spike (RBD Domain) of COVID-19 to nanomaterial, protein ferritin nanocages. Biomolecules. 2021;11(2):297. 33671255 10.3390/biom11020297 PMC7923090

[38] Walls AC, Park YJ, Tortorici MA, Wall A, McGuire AT, Veesler D. Structure, function, and antigenicity of the SARS-CoV-2 spike glycoprotein. Cell. 2020;181(2):281–292.e6. 32155444 10.1016/j.cell.2020.02.058 PMC7102599

[39] Wang B, Li S, Qiao Y, Fu Y, Nie J, Jiang S, Yao X, Pan Y, Zhao L, Wu C, et al. Self-assembling ferritin nanoparticles coupled with linear sequences from canine distemper virus haemagglutinin protein elicit robust immune responses. J Nanobiotechnol. 2022;20(1):32. 10.1186/s12951-021-01229-0 PMC8744384 35012571

[40] Attarilar S, Yang J, Ebrahimi M, Wang Q, Liu J, Tang Y, Yang J. The toxicity phenomenon and the related occurrence in metal and metal oxide nanoparticles: a brief review from the biomedical perspective. Front Bioeng Biotechnol. 2020;8:822. 32766232 10.3389/fbioe.2020.00822 PMC7380248

[41] Kelly HG, Tan H-X, Juno JA, Esterbauer R, Ju Y, Jiang W, Wimmer VC, Duckworth BC, Groom JR, Caruso F, et al. Self-assembling influenza nanoparticle vaccines drive extended germinal center activity and memory B cell maturation. Jci Insight. 2020;5(10):e136653. 32434990 10.1172/jci.insight.136653 PMC7259527

[42] Zhang XD, Chen XK, Zhao YL. Nanozymes: versatile platforms for cancer diagnosis and therapy. Nano-Micro Lett. 2022;14(1):95. 10.1007/s40820-022-00828-2 PMC8986955 35384520

[43] Liang MM, Yan XY. Nanozymes: from new concepts, mechanisms, and standards to applications. Acc Chem Res. 2019;52(8):2190–2200. 31276379 10.1021/acs.accounts.9b00140

[44] Gao L, Zhuang J, Nie L, Zhang J, Zhang Y, Gu N, Wang T, Feng J, Yang D, Perrett S, et al. Intrinsic peroxidase-like activity of ferromagnetic nanoparticles. Nat Nanotechnol. 2007;2(9):577–583. 18654371 10.1038/nnano.2007.260

[45] Zhu XH, Du JX, Zhu D, Ren SZ, Chen K, Zhu HL. Recent Research on Methods to Improve Tumor Hypoxia Environment. Oxid Med Cell Longevity . 2020;2020:1–18. 10.1155/2020/5721258 PMC7725563 33343807

[46] Candelaria PV, Leoh LS, Penichet ML, Daniels-Wells TR. Antibodies Targeting the Transferrin Receptor 1 (TfR1) as direct anti-cancer agents. Front Immunol. 2021;12:607692. 33815364 10.3389/fimmu.2021.607692 PMC8010148

[47] Hestericova M, Heinisch T, Lenz M, Ward TR. Ferritin encapsulation of artificial metalloenzymes: engineering a tertiary coordination sphere for an artificial transfer hydrogenase. Dalton Trans. 2018;47(32):10837–10841. 30019062 10.1039/c8dt02224k

[48] Zhang L, Laug L, Münchgesang W, Pippel E, Gösele U, Brandsch M, Knez M. Reducing stress on cells with apoferritin-encapsulated platinum nanoparticles. Nano Lett. 2010;10(1):219–223. 20017497 10.1021/nl903313r

[49] Wang T, He J, Duan D, Jiang B, Wang P, Fan K, Liang M, Yan X. Bioengineered magnetoferritin nanozymes for pathological identification of high-risk and ruptured atherosclerotic plaques in humans. Nano Res. 2019;12(4):863–868.

[50] Kasyutich O, Ilari A, Fiorillo A, Tatchev D, Hoell A, Ceci P. Silver ion incorporation and nanoparticle formation inside the cavity of pyrococcus furiosus ferritin: structural and size-distribution analyses. J Am Chem Soc. 2010;132(10):3621–3627. 20170158 10.1021/ja910918b

[51] Peskova M, Ilkovics L, Hynek D, Dostalova S, Sanchez-Carnerero EM, Remes M, Heger Z, Pekarik V. Detergent-modified catalytic and enzymomimetic activity of silver and palladium nanoparticles biotemplated by Pyrococcus furiosus ferritin. J Colloid Interface Sci. 2019;537:20–27. 30415098 10.1016/j.jcis.2018.11.005

[52] Foglizzo V, Marchio S. Nanoparticles as physically- and biochemically-tuned drug formulations for cancers therapy. Cancers. 2022;14(10):2473. 35626078 10.3390/cancers14102473 PMC9139219

[53] Kang C, Sun Y, Zhu J, Li W, Zhang A, Kuang T, Xie J, Yang Z. Delivery of nanoparticles for treatment of brain tumor. Curr Drug Metab. 2016;17(8):745–754. 27469219 10.2174/1389200217666160728152939

[54] Bhushan B, Kumar SU, Matai I, Sachdev A, Dubey P, Gopinath P. Ferritin nanocages: a novel platform for biomedical applications. J Biomed Nanotechnol. 2014;10(10):2950–2976. 25992425 10.1166/jbn.2014.1980

[55] Alkhateeb AA, Connor JR. The significance of ferritin in cancer: anti-oxidation, inflammation and tumorigenesis. Biochim Biophys Acta. 2013;1836(2):245–254. 23891969 10.1016/j.bbcan.2013.07.002

[56] Li L, Fang CJ, Ryan JC, Niemi EC, Lebrón JA, Björkman PJ, Arase H, Torti FM, Torti SV, Nakamura MC, et al. Binding and uptake of H-ferritin are mediated by human transferrin receptor-1. Proc Natl Acad Sci U S A. 2010;107(8):3505–3510. 20133674 10.1073/pnas.0913192107 PMC2840523

[57] Chen H, Tan X, Han X, Ma L, Dai H, Fu Y, Zhang Y. Ferritin nanocage based delivery vehicles: from single-, co- to compartmentalized- encapsulation of bioactive or nutraceutical compounds. Biotechnol Adv. 2022;61:108037. 36152892 10.1016/j.biotechadv.2022.108037

[58] Waller LP, Deshpande V, Pyrsopoulos N. Hepatocellular carcinoma: a comprehensive review. World J Hepatol. 2015;7(26):2648–2663. 26609342 10.4254/wjh.v7.i26.2648 PMC4651909

[59] Yao X, Liu H, Zhang X, Zhang L, Li X, Wang C, Sun S. Cell Surface GRP78 Accelerated Breast Cancer Cell Proliferation and Migration by Activating STAT3. Plos One. 2015;10(5):e0125634. 25973748 10.1371/journal.pone.0125634 PMC4431800

[60] Liang M, Fan K, Zhou M, Duan D, Zheng J, Yang D, Feng J, Yan X. H-ferritin-nanocaged doxorubicin nanoparticles specifically target and kill tumors with a single-dose injection. Proc Natl Acad Sci U S A. 2014;111(41):14900–14905. 25267615 10.1073/pnas.1407808111 PMC4205604

[61] Macone A, Masciarelli S, Palombarini F, Quaglio D, Boffi A, Trabuco MC, Baiocco P, Fazi F, Bonamore A. Ferritin nanovehicle for targeted delivery of cytochrome C to cancer cells. Sci Rep. 2019;9(1):11749 31409839 10.1038/s41598-019-48037-z PMC6692331

[62] Wang C, Wang X, Zhang W, Ma D, Li F, Jia R, Shi M, Wang Y, Ma G, Wei W, et al. Shielding Ferritin with a Biomineralized Shell Enables Efficient Modulation of Tumor Microenvironment and Targeted Delivery of Diverse Therapeutic Agents. Adv Mater . 2022;34(5):2107150. 10.1002/adma.202107150 34897858

[63] Kurtzberg LS, Roth S, Krumbholz R, Crawford J, Bormann C, Dunham S, Yao M, Rouleau C, Bagley RG, Yu X-J, et al. Genz-644282, a Novel Non-Camptothecin Topoisomerase I Inhibitor for Cancer Treatment. Clin Cancer Res. 2011;17(9):2777–2787. 21415217 10.1158/1078-0432.CCR-10-0542

[64] Falvo E, Arcovito A, Conti G, Cipolla G, Pitea M, Morea V, Damiani V, Sala G, Fracasso G, Ceci P, et al. Engineered Human Nanoferritin Bearing the Drug Genz-644282 for Cancer Therapy. Pharmaceutics. 2020;12(10):992. 33092088 10.3390/pharmaceutics12100992 PMC7589674

[65] Bio M, Mahabubur KM, Lim I, Rajaputra P, Hurst RE, You Y. Singlet oxygen-activatable Paclitaxel prodrugs via intermolecular activation for combined PDT and chemotherapy. Bioorg Med Chem Lett. 2019;29(12):1537–1540. 30987891 10.1016/j.bmcl.2019.03.053

[66] Li R, Ma Y, Dong Y, Zhao Z, You C, Huang S, Li X, Wang F, Zhang Y. Novel Paclitaxel-Loaded Nanoparticles Based on Human H Chain Ferritin for Tumor-Targeted Delivery. ACS Biomater Sci Eng. 2019;5(12):6645–6654. 33423483 10.1021/acsbiomaterials.9b01533

[67] Ma Y, Li R, Dong Y, You C, Huang S, Li X, Wang F, Zhang Y. tLyP-1 peptide functionalized human H chain ferritin for targeted delivery of paclitaxel. Int J Nanomedicine. 2021;16:789–802. 33568906 10.2147/IJN.S289005 PMC7869709

[68] Pandolfi L, Bellini M, Vanna R, Morasso C, Zago A, Carcano S, Avvakumova S, Bertolini JA, Rizzuto MA, Colombo M, et al. H-ferritin enriches the curcumin uptake and improves the therapeutic efficacy in triple negative breast cancer cells. Biomacromolecules. 2017;18(10):3318–3330. 28886247 10.1021/acs.biomac.7b00974

[69] Kuruppu AI, Zhang L, Collins H, Turyanska L, Thomas NR, Bradshaw TD. An Apoferritin-based Drug Delivery System for the Tyrosine Kinase Inhibitor Gefitinib. Adv Healthc Mater. 2015;4(18):2816–2821. 26592186 10.1002/adhm.201500389

[70] Inoue I, Chiba M, Ito K, Okamatsu Y, Suga Y, Kitahara Y, Nakahara Y, Endo Y, Takahashi K, Tagami U, et al. One-step construction of ferritin encapsulation drugs for cancer chemotherapy. Nanoscale. 2021;13(3):1875–1883. 33439183 10.1039/d0nr04019c

[71] Jiang B, Chen X, Sun G, Chen X, Yin Y, Jin Y, Mi Q, Ma L, Yang Y, Yan X, et al. A natural drug entry channel in the ferritin nanocage. Nano Today. 2020; 35:100948.

[72] Zhang BL, Tang GH, He JY, Yan XY, Fan KL. Ferritin nanocage: a promising and designable multi-module platform for constructing dynamic nanoassembly-based drug nanocarrier. Adv Drug Delivery Rev . 2021;176:113892. 10.1016/j.addr.2021.113892 34331986

[73] Chen S, Liu Y, Zhu L, Meng D, Zhang L, Wang Q, Hu J, Wang D, Wang Z, Zhou Z, et al. Chaotrope-controlled fabrication of ferritin-salvianolic acid B- epigallocatechin gallate three-layer nanoparticle by the flexibility of ferritin channels. J Agric Food Chem. 2021;69(41):12314–12322. 34612625 10.1021/acs.jafc.1c01997

[74] Jiang B, Fang L, Wu KM, Yan XY, Fan KL. Ferritins as natural and artificial nanozymes for theranostics. Theranostics. 2020;10(2):687–706. 31903145 10.7150/thno.39827 PMC6929972

[75] Li Y, Liu G, Ma J, Lin J, Lin H, Su G, Chen D, Ye S, Chen X, Zhu X, et al. Chemotherapeutic drug-photothermal agent co-self-assembling nanoparticles for near-infrared fluorescence and photoacoustic dual-modal imaging-guided chemo-photothermal synergistic therapy. J Control Release. 2017;258:95–107. 28501673 10.1016/j.jconrel.2017.05.011

[76] Wang Z, Huang P, Jacobson O, Wang Z, Liu Y, Lin L, Lin J, Lu N, Zhang H, Tian R. Biomineralization-inspired synthesis of copper sulfide-ferritin nanocages as cancer theranostics. Acs Nano 2016;10(3):3453–3460. 26871955 10.1021/acsnano.5b07521 PMC5242369

[77] Li H, Zhang W, Ding L, Li XW, Wu Y, Tang JH. Prussian blue-modified ferritin nanoparticles for effective tumor chemo-photothermal combination therapy via enhancing reactive oxygen species production. J Biomater Appl. 2019;33(9):1202–1213. 30714472 10.1177/0885328218825175

[78] Zhang J, Zeng Y, Su M, Yu M, Zhang Y, Cheng H, Zheng H, Liu J, Wang X, Lei Z, et al. Multifunctional Ferritin Nanoparticles as Theranostics for Imaging-Guided Tumor Phototherapy. J Biomed Nanotechnol. 2019;15(7):1546–1555. 31196357 10.1166/jbn.2019.2788

[79] Zhen ZP, Tang W, Zhang WZ, Xie J. Folic acid conjugated ferritins as photosensitizer carriers for photodynamic therapy. Nanoscale. 2015;7(23):10330–10333. 25998995 10.1039/c5nr01833a PMC4885642

[80] Lam JKW, Chow MYT, Zhang Y, Leung SWS. siRNA Versus miRNA as therapeutics for gene silencing. Mol Ther Nucleic Acids. 2015;4:e252. 26372022 10.1038/mtna.2015.23 PMC4877448

[81] Li L, Muñoz-Culla M, Carmona U, Lopez MP, Yang F, Trigueros C, Otaegui D, Zhang L, Knez M. Ferritin-mediated siRNA delivery and gene silencing in human tumor and primary cells. Biomaterials. 2016;98:143–151. 27187278 10.1016/j.biomaterials.2016.05.006

[82] Chen H, Ma L, Dai H, Fu Y, Han X, Zhang Y. The construction of self-protective ferritin nanocage to cross dynamic gastrointestinal barriers with improved delivery efficiency. Food Chem . 2022;397:133680. 35963111 10.1016/j.foodchem.2022.133680

[83] Zhou Z, Sun G, Liu Y, Gao Y, Xu J, Meng D, Strappe P, Blanchard C, Yang R. A Novel Approach to Prepare Protein-proanthocyanidins Nano-complexes by the Reversible Assembly of Ferritin Cage. FSTR. 2017;23(2):329–337.

[84] Yang R, Tian J, Liu YQ, Yang ZY, Wu DD, Zhou ZK. Thermally Induced Encapsulation of Food Nutrients into Phytoferritin through the Flexible Channels without Additives. J Agric Food Chem. 2017;65(46):9950–9955. 29037043 10.1021/acs.jafc.7b03949

[85] Wang Z, Zhao Y, Zhang S, Chen X, Sun G, Zhang B, Jiang B, Yang Y, Yan X, Fan K, et al. Re-engineering the inner surface of ferritin nanocage enables dual drug payloads for synergistic tumor therapy. Theranostics. 2022;12(4):1800–1815. 35198074 10.7150/thno.68459 PMC8825595

[86] Mansourizadeh F, Alberti D, Bitonto V, Tripepi M, Sepehri H, Khoee S, Geninatti Crich S. Efficient synergistic combination effect of Quercetin with Curcumin on breast cancer cell apoptosis through their loading into Apo ferritin cavity. Colloids Surf B Biointerfaces. 2020;191:110982. 32220813 10.1016/j.colsurfb.2020.110982

[87] Chen H, Dai H, Zhu H, Ma L, Fu Y, Feng X, Sun Y, Zhang Y. Construction of dual-compartmental micro-droplet via shrimp ferritin nanocages stabilized Pickering emulsions for co-encapsulation of hydrophobic/hydrophilic bioactive compounds. Food Hydrocolloids. 2022;126:107443.

[88] Pang J, Feng X, Liang Q, Zheng X, Duan Y, Zhang X, Zhang J, Chen Y, Fan K, Gao L, et al. Ferritin-nanocaged ATP traverses the blood-testis barrier and enhances sperm motility in an asthenozoospermia model. Acs Nano. 2022;16(3):4175–4185. 35167250 10.1021/acsnano.1c10029

[89] Kim JW, Lee KK, Park KW, Kim M, Lee CS. Genetically modified ferritin nanoparticles with bone-targeting peptides for bone imaging. IJMS. 2021;22(9):4854. 34063731 10.3390/ijms22094854 PMC8125493

[90] Yao HC, Zhao WW, Zhang SG, Guo XF, Li Y, Du B. Dual-functional carbon dot-labeled heavy-chain ferritin for self-targeting bio-imaging and chemo-photodynamic therapy. J Mater Chem B. 2018;6(19):3107–3115. 32254345 10.1039/c8tb00118a

[91] Antonelli A, Sfara C, Battistelli S, Canonico B, Arcangeletti M, Manuali E, Salamida S, Papa S, Magnani M. New strategies to prolong the in vivo life span of iron-based contrast agents for MRI. Plos One. 2013;8(10):e78542. 24223101 10.1371/journal.pone.0078542 PMC3819506

[92] Valero E, Fiorini S, Tambalo S, Busquier H, Callejas-Fernández J, Marzola P, Gálvez N, Domínguez-Vera JM. In vivo long-term magnetic resonance imaging activity of ferritin-based magnetic nanoparticles versus a standard contrast agent. J Med Chem. 2014;57(13):5686–5692. 24901375 10.1021/jm5004446

[93] Mittleman DM. Twenty years of terahertz imaging Invited. Opt Express. 2018;26(8):9417–9431. 29715894 10.1364/OE.26.009417

[94] Yang Z, Tang D, Hu J, Tang M, Zhang M, Cui H‐L, Wang L, Chang C, Fan C, Li J, et al. Near-field nanoscopic terahertz imaging of single proteins. Small. 2021;17(3):2005814. 10.1002/smll.202005814 33306275

[95] Huang XL, Xue Y, Wu JL, Zhan Q, Zhao JM. MRI tracking of SPIO- and Fth1-labeled bone marrow mesenchymal stromal cell transplantation for treatment of stroke. Contrast Media Mol Imaging . 2019;2019:1–10. 10.1155/2019/5184105 PMC6735219 31531004

[96] Lv CY, Yin SH, Zhang XQ, Hu JW, Zhang T, Zhao GH. 16-Mer ferritin-like protein templated gold nanoclusters for bioimaging detection of methylmercury in the brain of living mice. Anal Chim Acta. 2020;1127:149–155. 32800118 10.1016/j.aca.2020.06.055

